# Mixed Neuroendocrine–Non‐Neuroendocrine Tumor of Bile Duct

**DOI:** 10.1002/kjm2.70038

**Published:** 2025-05-20

**Authors:** Lian Shen, Wei‐Nan Liu, Shuo Qi, Chang‐Jun Liu

**Affiliations:** ^1^ Department of Hepatobiliary Surgery The First Affiliated Hospital of Hunan Normal University Changsha Hunan China

Neuroendocrine tumors of the bile duct are exceedingly rare in clinical practice, accounting for approximately 0.19% of extrahepatic bile duct tumors. The prevalence of mixed neuroendocrine–non‐neuroendocrine neoplasms (MiNEN) of the bile duct is even lower. Herein, we report a case with a postoperative pathological diagnosis of MiNEN, aiming to provide clinicians with insights into the diagnostic approach and treatment strategies for this rare entity.

A 64‐year‐old female patient presented with jaundice affecting the skin and sclera for over 10 days. Her symptoms progressively worsened, accompanied by generalized pruritus, normochromic stools, and dark tea‐colored urine. Laboratory investigations revealed significantly elevated liver function parameters: total bilirubin (TBIL) 218.99 μmol/L, direct bilirubin (DBIL) 163.32 μmol/L, and indirect bilirubin (IBIL) 55.67 μmol/L. Liver enzymes were also elevated, with alanine aminotransferase (ALT) at 127.7 U/L and aspartate aminotransferase (AST) at 122.9 U/L. Tumor marker analysis showed elevated CA19‐9 levels at 102.57 U/mL, while neuron‐specific enolase (NSE) remained within normal limits. Abdominal computed tomography (CT) and magnetic resonance imaging (MRI) (Figure 1A–C) demonstrated a mass in the porta hepatis region, suggestive of cholangiocarcinoma. Positron emission tomography/computed tomography (PET/CT) (Figure 1D,E) revealed wall thickening of the common hepatic duct, upper segment of the common bile duct, and gallbladder duct area, accompanied by luminal narrowing and abnormally increased metabolic activity with further enhancement in the delayed phase, consistent with malignancy. Following percutaneous transhepatic cholangial drainage (PTCD) to reduce bilirubin levels, the patient underwent extrahepatic bile duct resection, cholecystectomy, partial hepatectomy, retroperitoneal lymph node dissection, and biliary‐enteric drainage reconstruction. Postoperative pathological examination (Figure 1F) confirmed a mixed neuroendocrine–non‐neuroendocrine tumor of the extrahepatic bile duct, comprising approximately 70% small cell neuroendocrine carcinoma and 30% moderately to well‐differentiated adenocarcinoma. Immunohistochemical analysis revealed focal positivity for carcinoembryonic antigen (CEA), partial positivity for synaptophysin (Syn) and chromogranin A (CgA), and Ki‐67 proliferation indices of 90% in the adenocarcinoma component and 50% in the neuroendocrine carcinoma component. The patient received long‐term Tegafur treatment postoperatively, with no evidence of recurrence at 8‐month follow‐up.

**FIGURE 1:**
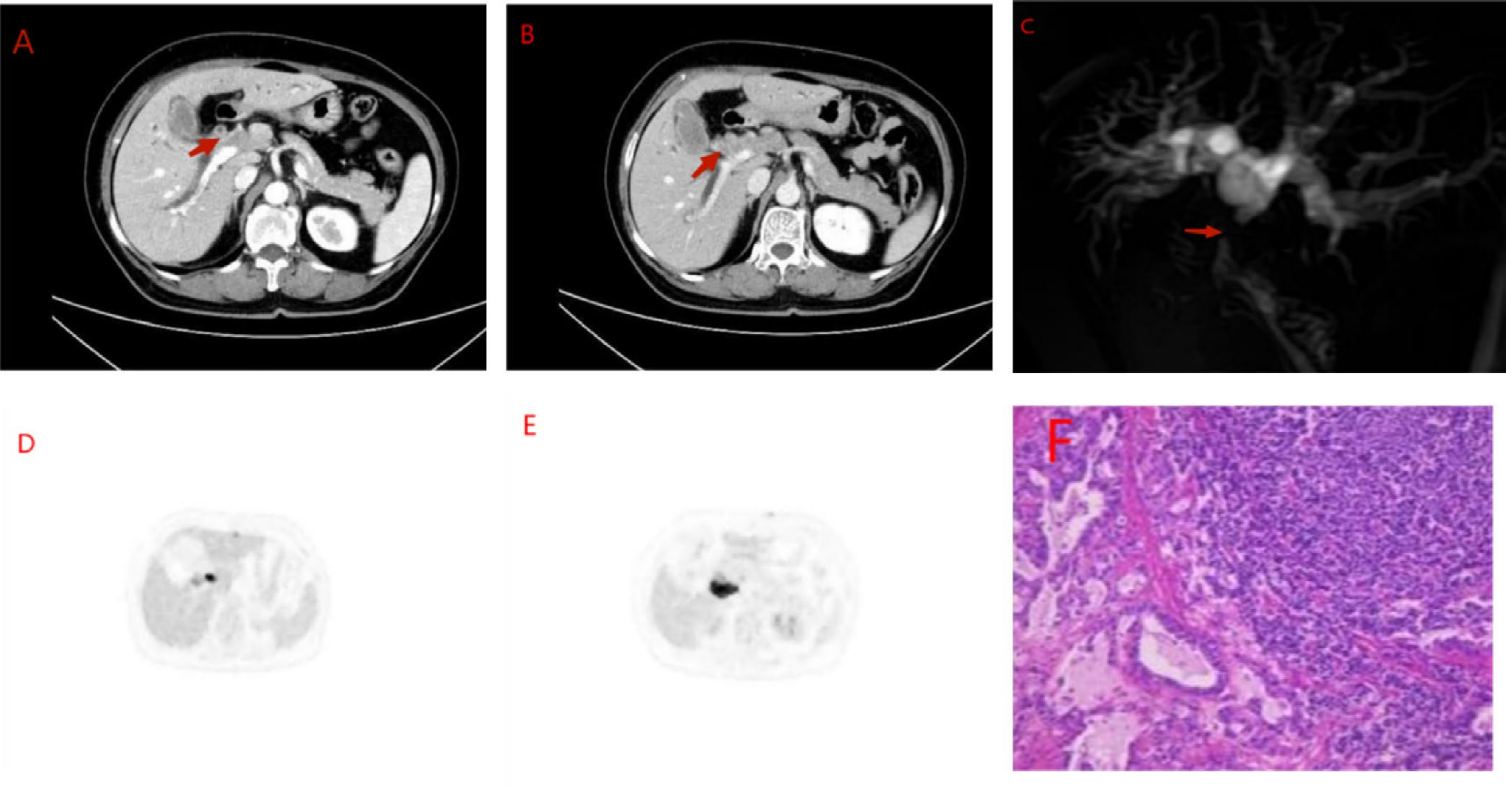
(A, B) CT enhanced showing arterial and venous phases reveals soft tissue density lesions in the hepatic hilum with significant continuous enhancement on post‐contrast sequences. (C) Magnetic resonance cholangiopancreatography (MRCP) demonstrates abrupt truncation of the common hepatic duct with a cup‐shaped filling defect in the lumen of the upper segment of the common bile duct. (D, E) PET/CT reveals wall thickening of the common hepatic duct, upper segment of the common bile duct, and cystic duct region with associated luminal stenosis. Abnormal radiotracer uptake is observed with further increased radiotracer concentration in the delayed phase, suggestive of malignancy. (F) Histopathological examination confirming the diagnosis of mixed MiNEN of the extrahepatic bile duct.

MiNEN refers to a mixed epithelial neoplasm containing both neuroendocrine and non‐neuroendocrine components, each distinguishable by histomorphology and immunohistochemistry, with each component constituting at least 30% of the tumor [1]. Definitive diagnosis of MiNEN primarily relies on postoperative histopathological and immunohistochemical assessment, with synaptophysin, insulinoma‐associated protein 1, and chromogranin A being the most sensitive biomarkers [2]. Patients with biliary MiNEN generally have a poor overall prognosis and limited survival, with reported median disease‐free survival of 5.3 months and overall survival of 12.2 months [3]. Surgical resection remains the cornerstone of treatment for biliary MiNEN. Depending on tumor location, procedures may include extrahepatic bile duct resection with reconstruction or pancreaticoduodenectomy [4]. Notably, Horita et al. [5] reported a case of unresectable extrahepatic biliary MiNEN that achieved complete remission following combination therapy with etoposide and cisplatin. This suggests that multimodal approaches incorporating preoperative chemotherapy, surgical resection, postoperative chemotherapy, and radiotherapy may potentially improve prognosis and survival outcomes for patients with biliary MiNEN.

## Conflicts of Interest

The authors declare no conflicts of interest.

## Data Availability

The data that support the findings of this study are available from the corresponding author upon reasonable request.
